# A systematic review and meta-analysis of carfilzomib-associated thrombocytopenia as an adverse event in patients with multiple myeloma

**DOI:** 10.1177/20406207241292517

**Published:** 2024-11-13

**Authors:** Lara Smrdel, Igor Locatelli, Samo Zver, Martina Gobec

**Affiliations:** Department of Clinical Biochemistry, Faculty of Pharmacy, University of Ljubljana, Ljubljana, Slovenia; Department of Biopharmaceutics and Pharmacokinetics, Faculty of Pharmacy, University of Ljubljana, Ljubljana, Slovenia; Clinical Department of Haematology, University Medical Centre Ljubljana, Ljubljana, Slovenia; Department of Clinical Biochemistry, Faculty of Pharmacy, University of Ljubljana, Aškerčeva cesta 7, Ljubljana SI-1000, Slovenia

**Keywords:** bortezomib, carfilzomib, multiple myeloma, proteasome, thrombocytopenia

## Abstract

**Background:**

Carfilzomib is a second-generation proteasome inhibitor (PI) used for combination therapy with dexamethasone and/or lenalidomide in patients with relapsed or refractory multiple myeloma. Reports indicate that PIs have a unique toxicity profile that includes thrombocytopenia as a hematologic adverse event; however, its occurrence has not yet been quantified systematically.

**Objectives:**

The main objective of our systematic review and meta-analysis is to investigate the incidence of thrombocytopenia in patients with multiple myeloma after treatment with carfilzomib.

**Design:**

Selection of studies and meta-analysis of trials was performed in accordance with the Preferred Reporting Items for Systematic Reviews and Meta-Analyses statement.

**Data sources and methods:**

Two investigators performed an independent literature search of PubMed, Web of Science, SciFinder, the Cochrane Central Register of Controlled Trials, as well as the US and EU clinical trials registries. The cumulative incidence and overall relative risk were calculated with the random effect model using RevMan and R statistical software.

**Results:**

The analysis included a total of 9237 patients, 2516 patients in single-arm studies and 6721 patients in randomized controlled trials (RCTs). A total of 47 studies were included; among these, 14 were RCTs. Analysis of currently available data showed that treatment with carfilzomib may increase the incidence of all-grade thrombocytopenia, and this correlated with the dose used. With supportive therapy alone, the incidence is 26%. The addition of carfilzomib to the treatment results in a 37% increase in incidence, whereas with bortezomib, the increase is slightly lower at 34%. Surprisingly, when treatment with carfilzomib and bortezomib was compared, bortezomib was found to be more likely to exacerbate high-grade thrombocytopenia (7%) than carfilzomib (3%).

**Conclusion:**

Clarification of these associations suggests that clinicians should be aware of the potential risk of high-grade thrombocytopenia occurring and monitor patients closely to take appropriate measures.

**Trial registration:**

Registered in PROSPERO under the number CRD42022314378.

## Plain language summary

Meta-analysis of carfilzomib-associated thrombocytopenia

**Background:**

Carfilzomib is a medication used in combination with other drugs for the treatment of relapsed or refractory multiple myeloma. Proteasome inhibitors, such as carfilzomib, are known to have certain side effects, including a decrease in platelet count (thrombocytopenia). However, the extent of this side effect has not yet been studied in detail.

**Objective:**

In this study, we have systematically reviewed and analysed existing studies to understand how common thrombocytopenia is in patients with multiple myeloma treated with carfilzomib.

**Methods:**

We followed established guidelines for conducting systematic reviews and meta-analyses. Our investigation included a thorough search of various medical databases and clinical trial registries.

**Results:**

A total of 47 studies, including 14 randomized controlled trials (RCTs), with a total of 9237 patients were included in the analysis. The results show that treatment with carfilzomib is associated with an increased incidence of thrombocytopenia. Specifically, the risk of thrombocytopenia of any grade is 26% with supportive therapy alone, but this risk increases to 37% with additional treatment with carfilzomib. Additionally, the dosage of carfilzomib appears to be a factor in this increased risk. A comparison of carfilzomib with another drug, bortezomib, showed that bortezomib is more likely to worsen severe thrombocytopenia (high-grade) compared to carfilzomib.

**Conclusion:**

In conclusion, our study underscores the importance of recognizing and monitoring the risk of thrombocytopenia, especially the more severe cases, in patients treated with carfilzomib. Clinicians should be vigilant and take appropriate measures to manage this potential side effect.

## Graphical abstract

**Visual abstract:**
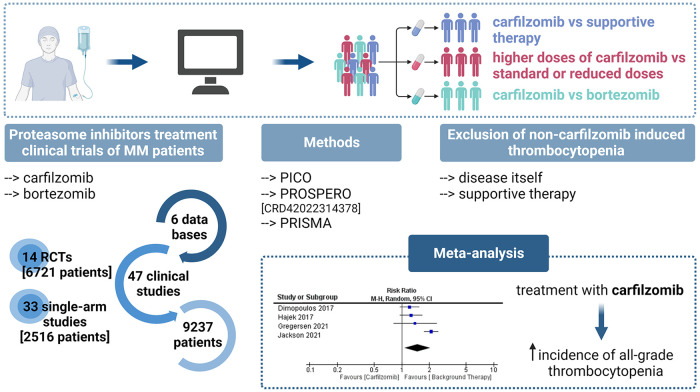

## Introduction

The prognosis for multiple myeloma (MM) patients has significantly improved with the introduction of immunomodulatory drugs and proteasome inhibitors (PIs) in the routine treatment.^1^ The first PI approved in 2003 was bortezomib,^2^ followed by the use of second-generation PIs due to reported development of treatment resistance and off-target effects of bortezomib.^2,3^ One of the newer generation PIs is carfilzomib, which has high selectivity toward two catalytically active subunits of the 20S proteasome—β5c and β5i.^3^ The carfilzomib-based treatment was approved by the FDA in 2012 for combination therapy with dexamethasone and/or lenalidomide for patients with relapsed or refractory MM (RRMM).^4^ However, PIs have shown serious adverse events (AEs) that are likely linked to inhibition of the ubiquitin-proteasome system, which is an important component of cellular signaling pathways that can lead to apoptosis.^5^ In addition, reports show that PIs have a unique toxicity profile that includes thrombocytopenia as a hematologic AE; however, this occurrence has not yet been directly associated with carfilzomib. Moreover, a direct comparison between PI-based therapeutic regimens has not yet been performed.

Data from four phase II clinical trials suggest that carfilzomib use in prior RRMM has a favorable safety profile. The most common AEs were both non-hematologic (fatigue and nausea) and hematologic (thrombocytopenia and anemia). The non-hematologic AEs were predominantly grade 1 or 2 in severity, whereas the hematologic AEs were grade 3 or 4 in severity and occurred in 80.2% of patients.^6^ These results confirm data from the systematic review of phase II clinical trials of carfilzomib for the treatment of MM, in which the incidence of thrombocytopenia as AE was reported in 25% and neutropenia in 12% of patients.^7^ A similar trend in AEs was observed in phase III clinical trials of carfilzomib in RRMM patients, with the most common AEs being neutropenia (30%), anemia (18%), thrombocytopenia (17%), and some non-hematologic AEs.^8^ The acceptability of carfilzomib treatment is confirmed by the low proportion of patients who had to discontinue or reduce treatment because of AEs. In addition, the collected data show that the proportion of patients who discontinued clinical trials with bortezomib because of safety concerns was higher than with carfilzomib. More information about the differences between the two PIs is provided by the results of the phase III clinical trials (ENDEAVOR and CLARION), in which a direct comparison between these two groups is possible.^6^ However, clinical trials of carfilzomib-based combination therapies have shown a signal of decreased platelet count or thrombocytopenia. Nevertheless, there is a lack of evidence to support the assessment of all-grade and severe or high-grade toxicities with carfilzomib-based combination therapies compared with non-carfilzomib therapies. As MM evolves into a chronic disease requiring continuous therapy, an adequate understanding of the adverse effects of therapy is critical for management.

The main objective of our systematic review and meta-analysis is to investigate the frequency of thrombocytopenia or decreased platelet count in patients with MM treated with carfilzomib.

## Methods

### Study guideline

The selection of studies and systematic review of trials was performed in accordance with the Preferred Reporting Items for Systematic Reviews and Meta-Analysis (PRISMA, 2020) statement.^9^ The protocol for this meta-analysis was registered with the International Prospective Register for Systematic Reviews or PROSPERO (number CRD42022314378).

### Ethics statement

Due to the meta-analytical nature of this study and the use of aggregated patient information, ethical approval was not required.

### Literature search strategy

The literature used for the systematic review and further meta-analysis was defined according to the structured PICOS principle (P: population, I: intervention, C: comparison, O: outcome, S: study design). Patients with MM, that is, RRMM or newly diagnosed multiple myeloma (NDMM), were selected as the target population (age 18+). The intervention studied is carfilzomib treatment; the control group (if present) could be supportive therapy, bortezomib treatment, or different carfilzomib dosing. The main outcome was the incidence of thrombocytopenia or decreased platelet count. The primary study design was randomized control trial; however, single-arm studies with carfilzomib therapy were also included.

The keywords of the search profiles used were carfilzomib (Kyprolis, PR-171, PR171), thrombocytopenia, cancer, and MM. The specific search profiles for the databases and registries used are listed in the supplementary material. Two investigators conducted an independent literature search of PubMed, Web of Science, SciFinder, and the Cochrane Central Register of Controlled Trials. We also searched the US (http://ClinicalTrials.gov) and EU (https://www.clinicaltrialsregister.eu) clinical trial registries. The following filters were used in the literature search: phase I/II or II or III clinical trials, publication date between January 2011 and January 2022, and English language. In the case of multiple publications available for the same clinical trial, the most recent publication was used to access the effect. If detailed data on AEs were published in the US or EU Clinical Trials Register, these data were also included. Studies that met the following criteria were considered: Adults (over 18 years of age) treated with carfilzomib- or bortezomib-based therapy for newly diagnosed or relapsed/refractory MM. Following were the exclusion criteria: adolescents (under 18 years of age) and individuals with solid tumors such as small cell lung, non-small cell lung, renal or ovarian cancer, systemic light chain amyloidosis, or other monoclonal gammopathies of clinical significance that do not meet the criteria for MM.

### Selection of studies and data acquisition

Two investigators performed data extraction independently, and any discrepancies were resolved by consensus. We recorded the total number of patients in the study and control arms, various disease-related factors, the dosing regimen for carfilzomib and supportive therapy (e.g., dexamethasone, lenalidomide, melphalan, pomalidomide, and cyclophosphamide), the degree of thrombocytopenia and neutropenia as defined in the Common Terminology Criteria for Adverse Events (CTCAE). All-grade and high-grade (⩾3) events were assessed separately for thrombocytopenia.

### Subgroup analysis

A subgroup meta-analysis was performed to evaluate the impact of different risk factors on the incidence of thrombocytopenia. The obtained randomized control trials (RCTs) were divided into three groups, namely: (1) carfilzomib compared with supportive therapy (e.g., dexamethasone, lenalidomide, melphalan, pomalidomide, and cyclophosphamide); (2) higher doses of carfilzomib (45, 56, 70, and 88 mg/m^2^) compared with standard (27 and 36 mg/m^2^) or reduced doses (15 and 20 mg/m^2^); and (3) carfilzomib compared with bortezomib treatment. Finally, thrombocytopenia incidence was also evaluated in the single-arm clinical trials in which carfilzomib treatment was combined with supportive therapy only.

### Statistical analysis of data

The studies used had dichotomous outcomes (occurrence of thrombocytopenia), so the number of participants with an event, the number of participants without an event, and the sample size included in the analysis were recorded for each intervention group. The effect size was expressed as relative risk (RR) with 95% CI. All tests with a value of *p* < 0.05 were considered statistically significant. Heterogeneity between studies was assessed using the statistical test *I*^2^. A random-effects model was considered due to expected huge variability. Data was managed and analyzed using a MS Excel 2019 (Microsoft Corporation, Redmond, WA, USA), RevMan 5.4.1 (Cochrane, London, UK, 2020), and R Statistical Software (v4.1.2; R Core Team, R Fundation for Statistical Computing, Vienna, Austria, 2021) via “meta” R package (v5.5-0).

## Results

**Figure 1.**
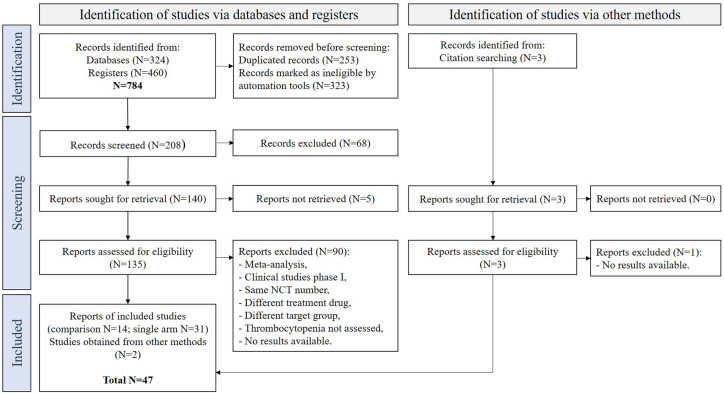
Process of selection of clinical trials for meta-analysis—PRISMA flowchart.

To our knowledge, this is the first meta-analysis to examine thrombocytopenia associated with carfilzomib treatment in MM patients. The analysis included a total of 9237 patients, including 2516 patients in single-arm studies and 6721 patients in RCTs. A total of 47 studies were included, among these 14 RCTs. Of the 137 records obtained after screening, 90 were excluded because they were not phase I/II, II, or III RCT or single-arm clinical trials, used a different treatment drug or target population, and lacked data on the incidence of thrombocytopenia, and results were not available. The complete stepwise clinical trial selection process is described in Figure 1.

### The RCTs study characteristics

In RCTs^10–24^ carfilzomib therapies were divided in four subgroups comparing (1) carfilzomib with background therapy (1142 patients vs 1146 patients), (2) reduced with standard carfilzomib doses (400 patients vs 160 patients), (iii) standard with high carfilzomib doses (325 patients vs 340 patients), and (iv) carfilzomib with bortezomib treatment (1659 patients vs 1549 patients). The median age of patients enrolled in the RCT studies ranged between 57^21^ and 72 years (CLARON study^13^). Characteristics of included RCT studies are listed in the Table 1. Of the 14 studies, 11 included patients with RRMM, and 3 included patients with NDMM. We have focused only on patients with decreased platelet count or thrombocytopenia referred to as AE (all-grade or high grade). In this systematic review and meta-analysis, clinical trials with patients having either Waldenstrom’s macroglobulinemia or solid tumors such as small cell or non-small cell lung cancer, kidney and ovarian cancer, are not included.

**Table 1.** Characteristics of the RCT studies included in meta-analysis.

Author, Year of publication, Study ID	Phase	Patients	Treatment regimen	Carfilzomib dosing (mg/m^2^)	Age (median)	Comparison group	NCT number
Gregersen 2021, CARFI	II	RMM	Carf, Cyc, Dex	20 (days 1 and 2) f/b 36 (days 8, 9, 15, and 16) f/b 36 (days 1, 2, 8, 9, 15, and 16)	62	Carf vs Support	NCT02572492
Jackson 2021, Myeloma XI+	III	NDMM	Carf, Len, Dex, Cyc	20 (days 1 and 2) f/b 36 (days 1, 2, 8, 9, 15, and 16)	62	Carf vs Support	ISRCTN49407852
Mehta 2021, —	—	RRMM	Carf, Pom, Dex	Biweekly regimen—20 (days 1 and 2) f/b 27 (days 8, 9, 15, and 16) or Once weekly regimen—20 (days 1 and 2) f/b 36 (days 8, and 15)	57	Standard vs High dose	—
Ailawadhi 2020, S1304	II	RRMM	Carf, Dex	Low-dose 27 (days 1, 2, 8, 9, 15, and 16) vs high-dose 56 (days 1, 2, 8, 9, 15, and 16)	⩾ 65	Standard vs High dose	NCT01903811
Kumar 2020, EDURANCE	III	NDMM	Carf, Btz, Dex, Len	36 (days 1, 2, 8, 9, 15, and 16)	64.5	Carf vs Btz	NCT01863550
Facon 2019, CLARION	III	NDMM	Carf, Btz, Mel, Pred	20 (days 1, 2) f/b 36 (days 8, 9, 22, 23, 29, and 30)	71.7	Carf vs Btz	NCT01818752
Ludwig 2019, ENDEAVOR	III	RMM	Carf, Btz, Dex	20 (days 1, 2) f/b 56 (days 8, 9, 15, and 16)	65	Carf vs Btz	NCT01568866
Moreau 2019, ARROW	III	RRMM	Carf, Dex	once weekly 20 (day 1) f/b 70 (days 8 and 15) or twice weekly 20 (days 1 and 2) f/b 27 (days 8, 9, 15, and 16)	66	Standard vs High dose	NCT02412878
Dimopoulos 2017, ASPIRE	III	RMM	Carf, Len, Dex	20 (days 1 and 2) f/b 27 (days 8, 9, 15, and 16) f/b (days 1, 2, 8, 9, 15, 16) f/b 27 (days 1, 2, 15, and 16)	63.9	Carf vs Support	NCT01080391
Hajek 2017, FOCUS	III	RRMM	Carf	20 (days 1 and 2) f/b 27 (days 8, 9, 15, and 16) f/b (days 1, 2, 8, 9,15, and 16) f/b 27 (days 1, 2, 15, and 16)	64.4	Carf vs Support	NCT01302392
Brown 2016, MUK five	II	RRMM	Carf, Btz, Dex, Cyc	20 (days 1 and 2) f/b 36 (days 1, 2, 8, 9, 15, and 16)	67	Carf vs Btz	ISRCTN17354232
Wang 2013, PX-171-006	II	RMM	Carf, Len, Dex	15, 20, or 20/27 (days 1, 2, 8, 9, 15, and 16)	61.9	Reduced vs Standard dose	NCT00603447
Jagannath 2012 & Siegel 2012, PX-171-003-A1	II	RRMM	Carf	20 (days 1, 2, 8, 9, 15, and 16) A0 vs 20 (days 1, 2, 8, 9, 15, and 16) f/b 27 (days 1, 2, 8, 9, 15, and 16)	60–65	Reduced vs Standard dose	NCT00511238
Vij 2012, PX-171-004	II	RMM	Carf	20 (days 1, 2, 8, 9, 15, and 16) f/b 27	65	Reduced vs Standard dose	NCT00530816

Btz, bortezomib; Carf, carfilzomib; Cyc, cyclophosphamide; Dex, dexamethasone; Len, lenalidomide; Mel, melphalan; NDMM, newly diagnosed multiple myeloma; NR, no results; Pred, prednisone; Pom, pomalidomide; RCT, randomized controlled trial; RRMM, relapse/refractory multiple myeloma.

Table 1 provides detailed characteristics of the RCTs, including treatment regimen information. Each RCT has a different study design due to the different choice of background therapy, carfilzomib dose (reduced, standard, or high dose), treated patients (RRMM and NDMM data combined), and characteristics of the study arms. The “Treatment Regimen” section lists carfilzomib and bortezomib treatment as well as background therapy, which can be divided into three drug groups: steroids (dexamethasone, prednisone), immunomodulatory treatment (lenalidomide, pomalidomide), and cytostatic treatment (cyclophosphamide, melphalan). Table 1 also provides information on the detailed dosing regimens for carfilzomib treatment, as well as the median age and comparison group defined according to the treatment regimen in each study arm.

### Incidence of thrombocytopenia in RCTs

The cumulative incidence of all-grade and high-grade thrombocytopenia was calculated from 14 RCTs involving 6721 patients (listed in Table 2). The incidence of all-grade thrombocytopenia ranged from 5% to 85% (mean: 37%) of patients treated with carfilzomib, including patients who received a reduced dose. In addition, the incidence of all-grade thrombocytopenia was slightly lower in the compared groups, ranging from 2% to 68% (mean: 34%) in patients treated with bortezomib and from 21% to 35% (mean: 26%) in patients receiving supportive therapy only. On the other hand, the incidence of high-grade thrombocytopenia was from 0% to 15% (mean: 3%) in patients treated with carfilzomib, 1%–22% (mean: 7%) in patients treated with bortezomib, and from 1% to 3% (mean: 2%) in patients receiving supportive therapy only. However, the MUK five trial,^11^ in which patients received both cyclophosphamide and lenalidomide as supportive therapy, had a higher incidence of both all-grade and high-grade thrombocytopenia, whereas the Myeloma XI+^16^ and CARFI^14^ trials, in which patients received cyclophosphamide, did not differ significantly from other trials. This could be a consequence of the use of lenalidomide, as this immunomodulatory drug is known to cause leukopenia, neutropenia, anemia, and thrombocytopenia as common AE.^25^

**Table 2.** Incidence of thrombocytopenia in RCT studies based on comparison.

Author, Year of publication	Phase	Total *N*	Carfilzomib treatment			Comparison group			Comparison group definition
			All-grade	High grade	*N*_scr._	All-grade	High grade	*N*_scr._	
Gregersen 2021	II	200	24	0	82	18	2	86	Carf vs Support
Jackson 2021	III	1029	256	43	511	124	9	518	Carf vs Support
Mehta 2021	—	69	9	4	30	6	1	39	Standard vs High dose
Ailawadhi 2020	II	143	32	4	66	36	7	57	Standard vs High dose
Kumar 2020	III	1087	26	3	526	11	4	527	Carf vs Btz
Facon 2019	III	955	139	7	474	166	14	470	Carf vs Btz
Ludwig 2019	III	929	163	6	463	133	9	456	Carf vs Btz
Moreau 2019	III	478	62	5	238	45	5	235	Standard vs High dose
Dimopoulos 2017	III	792	115	5	392	94	4	389	Carf vs Support
Hajek 2017	III	315	70	3	157	54	5	153	Carf vs Support
Brown 2016	II	292	167	22	196	65	21	96	Carf vs Btz
Wang 2013	II	84	8	0	20	22	1	64	Reduced vs Standard dose
Siegel 2012 and Jagannath 2012	II	312	23	0	46	103	5	266	Reduced vs Standard dose
Vij 2012	II	164	31	1	94	19	0	70	Reduced vs Standard dose

Btz, bortezomib; Carf, carfilzomib; *N*_scr_., number of patients screened for thrombocytopenia occurrence; RCT, randomized controlled trial; Support, supportive therapy.

### Carfilzomib and high-grade thrombocytopenia

**Figure 2.**
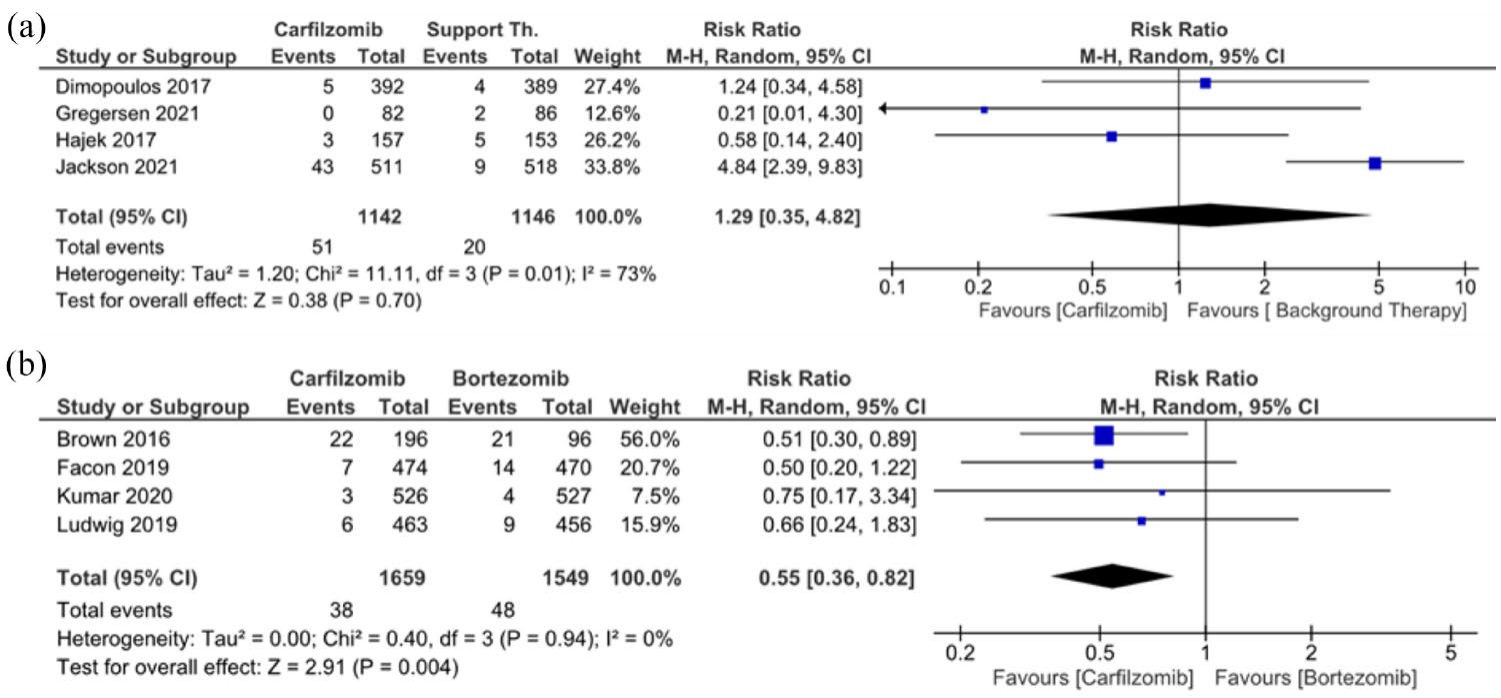
Incidence of high-grade thrombocytopenia in RCTs. (a) Patients receiving carfilzomib treatment versus patients receiving supportive therapy only. (b) Patients receiving carfilzomib versus patients receiving bortezomib treatment. RCT, randomized controlled trial.

In contrast to all-grade thrombocytopenia, we found no significant difference in the RR of high-grade thrombocytopenia between patients in the carfilzomib and supportive therapy groups (*p* = 0.70). A higher incidence of high-grade thrombocytopenia in the control group was observed in two of four studies with smaller sample sizes, but the weight of the studies was comparable to studies with larger sample sizes. As mentioned earlier, the lower incidence of high-grade thrombocytopenia observed in the CARFI trial^14^ may be a consequence of supportive therapy with cyclophosphamide. Heterogeneity between studies is also high, but the trend is in favor of supportive therapy, with an RR of 1.29 (95% CI: 0.35, 4.82; Figure 2(a)), indicating that high-grade thrombocytopenia was associated with carfilzomib treatment in the ASPIRE^12^ and Myeloma XI+^16^ study.

A significant difference was observed when carfilzomib and bortezomib treatment were compared. Results from MUK five,^11^ CLARION,^13^ EDURANCE,^19^ and ENDEAVOR^20^ showed that bortezomib was more likely to cause high-grade thrombocytopenia than carfilzomib, with an RR of 0.55 (95% CI: 0.36, 0.82, *p* = 0.004), Figure 2(b). Lonial et al. observed high-grade thrombocytopenia (grade 3/4) associated with bortezomib treatment. In patients receiving bortezomib, grade 3 thrombocytopenia occurred in 26% and grade 4 in 4%, whereas in patients receiving dexamethasone, grade 3 thrombocytopenia occurred in only 5% and grade 4 in 1%.^26^

**Figure 3.**
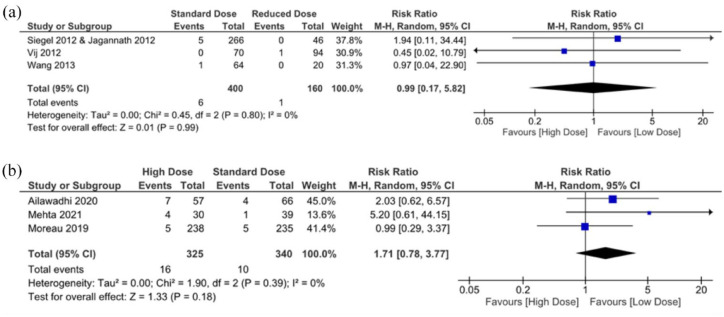
Incidence of high-grade thrombocytopenia in RCTs. (a) Patients receiving standard dose carfilzomib treatment versus patients receiving reduced dose treatment. (b) Patients receiving high dose carfilzomib treatment versus patients receiving standard dose treatment. RCT, randomized controlled trial.

When the reduced carfilzomib dose was compared to the standard dose, there was no significant difference (*p* = 0.99; Figure 3(a)). This is not unexpected as the carfilzomib doses received were not significantly different, that is, the standard doses were considered 27 and 36 mg/m^2^ and the reduced doses were 15 and 20 mg/m^2^. However, there was also no significant difference between patients who received a standard and patients who received a high dose of carfilzomib (*p* = 0.18). Nonetheless, there is an implied trend for the standard dose of carfilzomib treatment, suggesting that higher doses of carfilzomib may increase high-grade thrombocytopenia with an RR of 1.71 (95% CI: 0.78, 3.77; Figure 3(b)), but not significantly.

### Carfilzomib and all-grade thrombocytopenia

We divided the data from the RCTs and performed a meta-analysis for several subgroups. There was no significant difference in the RR for the occurrence of all-grade thrombocytopenia between patients treated with carfilzomib and those treated with bortezomib (*p* = 0.24). No significant difference was also between patients treated with reduced and standard doses of carfilzomib (*p* = 0.08). In this case, the studies show no heterogeneity, and the trend line is slightly on the side of the standard dose, suggesting that the reduced dose of carfilzomib is more likely to cause all-grade thrombocytopenia than the standard dose. This may be a consequence of the small sample size (only 560 patients were included) and the more aggressive supportive therapy after the reduced carfilzomib dose since Wang et al. reported additional treatment with lenalidomide and dexamethasone in combination with carfilzomib in the control group.^24^ However, the RR of all-grade thrombocytopenia between patients treated with standard and high doses of carfilzomib is significantly higher in the subgroup in which patients received higher doses (*p* = 0.007). Therefore, a favorable subgroup is patients receiving standard therapy with carfilzomib, with an RR of 1.36 (95% CI: 1.09, 1.70) and little heterogeneity between RCTs. More important, however, were the data between the subgroup receiving carfilzomib and the group receiving supportive therapy only. We found a significant difference between these two groups, with the carfilzomib-treated group having a significantly higher RR of all-grade thrombocytopenia (*p* = 0.02). The higher RR of thrombocytopenia cannot be attributed to the use of lenalidomide (ASPIRE^12^) and dexamethasone (all four RCTs), as both groups received the same drugs as supportive therapy. The RR was 1.47 (95% CI: 1.07, 2.04), and a random effect model was used because of the large heterogeneity between studies.

### Incidence of thrombocytopenia in single-arm studies

**Figure 4.**
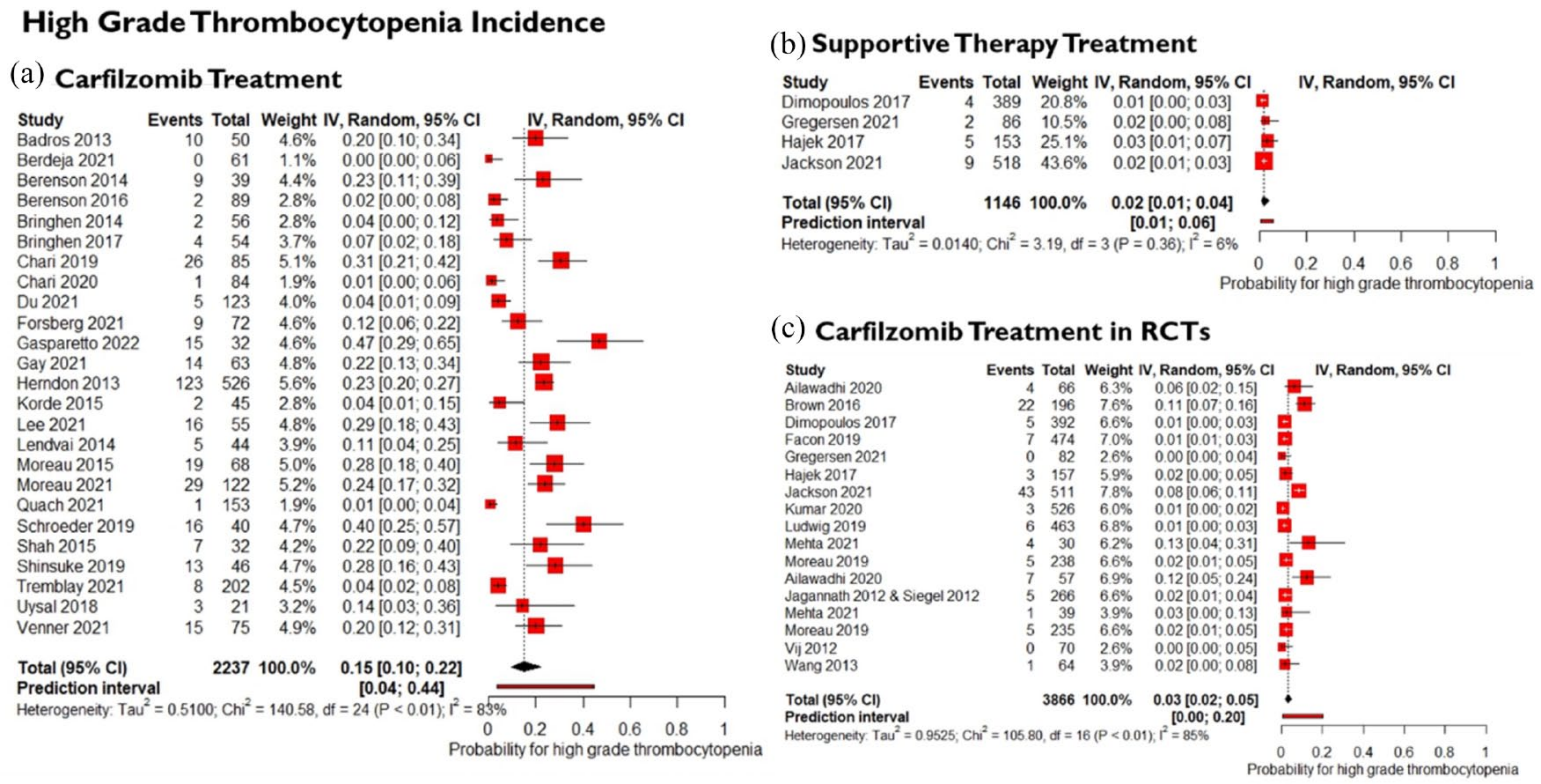
Incidence of high-grade thrombocytopenia in single-arm studies. (a) Patients receiving carfilzomib treatment. (b) Patients receiving supportive therapy only. (c) Patients receiving standard dose of carfilzomib treatment in RCTs. RCT, randomized controlled trial.

The cumulative incidence of all-grade thrombocytopenia in 2561 patients treated with carfilzomib from 31 studies was 60% (95% CI: 50, 69). We also calculated the cumulative incidence of all-grade thrombocytopenia among 1146 patients receiving supportive therapy only from four RCTs, and it was 26% (95% CI: 18, 36). Comparing these two outcomes we can see that incidence of thrombocytopenia is 2.3-fold higher in carfilzomib treatment arm compared to supportive therapy control group used in RCTs. Also, in single-arm studies the data is more scattered and heterogenous than in RCTs, where the predictive interval is 22%–89% compared to 11%–51%, respectively. Discussed data and forest plots are accessible in supplementary material. In addition, the cumulative incidence of high-grade thrombocytopenia was calculated, and only 25 studies were included due to missing data. The incidence of high-grade thrombocytopenia in 2237 patients treated with carfilzomib was 15% (95% CI: 10–22); however, the prediction interval is very wide (4%–44%) and marked heterogeneity (*I*^2^ = 83%) among the studies is present, Figure 4(a). Furthermore, the heterogeneity could not be explained by carfilzomib treatment dose nor by the medicines in the supportive therapy. On the other hand, incidence of high-grade thrombocytopenia in 1146 patients from 4 RCTs who received supportive therapy only was significantly lower, that is, 2% (95% CI: 1, 4), Figure 4(b). The cumulative incidence and heterogeneity are approximately sevenfold higher in single-arm studies than in RCTs. In addition, we calculated the incidence of high-grade thrombocytopenia using data from RCTs patients (Figure 4(c)). Calculated incidence in 3866 patients receiving standard dose (20, 27, and 36 mg/m^2^) of carfilzomib treatment was 3% (95% CI: 2, 5), showing that standard doses of carfilzomib treatment may not increase the incidence of high-grade thrombocytopenia. Figure 4(c) shows that data acquired in RCTs is more homogenous than single-arm studies data due to more regulated conduction instructions and randomization of participants in clinical trials.

## Discussion

In our meta-analysis, we analyzed RCTs focusing on carfilzomib therapies, and divided them into four different subgroups. These subgroups included trials evaluating carfilzomib with supportive therapy, comparing reduced versus standard carfilzomib doses, standard versus high carfilzomib doses, and assessing carfilzomib versus bortezomib treatment. Our analysis was specific to patients with RRMM and NDMM who experienced decreased platelet count or thrombocytopenia as AEs. The supportive therapies used in these studies were categorized into three main groups: steroids (dexamethasone, prednisone), immunomodulatory treatment (lenalidomide, pomalidomide), and cytostatic treatment (cyclophosphamide, melphalan).

On the one hand, steroids such as dexamethasone (20–40 mg) and prednisone (60 mg/m^2^) play a central role in alleviating the side effects of chemotherapy by reducing inflammation and nausea, and suppressing the immune response.^27^ In addition, steroids exhibit a strong proapoptotic impact on MM cell lines and contribute significantly to the effectiveness of the Vincristine-Adriamycine-Dexamethasone (VAD) chemotherapy regimen.^28^ On the other hand, immunomodulatory and cytostatic treatments target cancer cells by inhibiting tumor growth, having an antiangiogenic effect, and causing cell death.^27^ In addition, lenalidomide was used in four RCTs (25 mg), pomalidomide in one RCT (4 mg), cyclophosphamide in three RCTs (500 mg), and melphalan in one RCT (9 mg/m^2^). Despite the different mechanisms of action, there were no significant differences in the dosage of the therapeutic agents used in RCTs. However, it is crucial to acknowledge that the aforementioned supportive treatments could potentially contribute to the occurrence of thrombocytopenia, as they are known to cause neutropenia. This consideration underscores the complexity of treatment regimens in MM patients and highlights the need for comprehensive monitoring and management strategies to effectively address hematologic toxicities.

Hematologic toxicities, both thrombocytopenia and neutropenia, are known to be common complications of various chemotherapy regimens in patients with MM.^26,29^ These patients have a clonal plasma cell malignancy accompanied by extensive myelophthisis, disruption of bone marrow stroma, and, to a lesser extent, elevated plasma viscosity, which not only affects platelet count but also influences their functionality.^30^ Thrombocytopenia is primarily linked to the cytotoxic effect of treatment on megakaryocytes, while neutropenia stems from hematopoietic disruption in the bone marrow. The decreased platelet count increases the risk of bleeding, necessitating careful management strategies. Rather than immediately adjusting or withholding carfilzomib dosage, it is suggested that an alternative PI could be administered at an equivalent doses, along with concomitant platelet supplementation if necessary.^26,29^ This approach aims to mitigate thrombocytopenia-related complications without compromising treatment efficacy.

Furthermore, it should be noted that MM patients may already have greater than 50% plasmacytosis in the bone marrow before treatment with carfilzomib, which indicates pre-existing cytopenia.^31^ Monitoring of cytopenia during treatment revealed that high-grade thrombocytopenia could be a consequence of carfilzomib treatment, which was particularly evident by the occurrence of neutropenia in several RCTs.

Most RCTs reported a minor incidence of high-grade neutropenia (<3%), with the exception of three RCTs: MUK five,^11^ Myeloma XI+,^16^ and CARFI,^14^ where incidence rates ranged from 5% to 20%. Interestingly, differences in neutropenia rates were observed depending on the supportive therapies, such as cyclophosphamide dosing. In MUK five,^11^ the incidence of high-grade neutropenia was highest in the bortezomib arm (21.9%) but lower by almost half in the carfilzomib arm (11.2%). Myeloma XI+^16^ had a more consistent incidence (approx. 17%) in both study arms which received both cyclophosphamide and lenalidomide. In the patients described in the CARFI study,^14^ the incidence of high-grade neutropenia was not as high (about 5%) because the cyclophosphamide dose was 300 mg/m^2^, whereas the dose was higher in the other two studies.

These findings suggest that high-grade neutropenia may serve as an exclusion criterion for thrombocytopenia being solely due to the disease itself. This is particularly relevant for NDMM patients as disease progression leads to increased heterogeneity due to various cofactors, such as stromal cell involvement.^32^ Therefore, frequent monitoring of platelet count during carfilzomib treatment initiation is needed. Overall, while carfilzomib therapy increases the risk of thrombocytopenia, the greatest risk for maintaining an adequate platelet count lies in severity of the disease itself, as reflected by the number of cycles of carfilzomib therapy received. This underscores the necessity for a balanced and personalized approach to treatment management.

High-grade thrombocytopenia, defined as a platelet count below 10,000/µl (in some cases below 20,000/µl), is a threshold for prophylactic platelet pool transfusions. However, the routine use of platelet transfusions is not recommended due to the risk of allosensitization to various platelet antigens, especially in the absence of bleeding indications. It is recommended to continue treatment at the previous dose, with the exception of patients recovering to grade 3, where a reduction of one dose level is recommended. Once the platelet count has stabilized, the carfilzomib dose can be increased back to the prescribed level. Nevertheless, severe thrombocytopenia accompanied by major bleeding is also an indication for discontinuation of carfilzomib therapy. In addition, MM patients undergoing carfilzomib treatment should be educated on identifying and reporting signs and symptoms of thrombocytopenia, such as petechiae, unexpected bruising, severe headaches, nosebleeds or bleeding gums, increased fatigue, and dizziness.^31^ This awareness enables patients to communicate effectively with healthcare providers, facilitating timely intervention and treatment of complications associated with thrombocytopenia.

In our analysis, we found no significant difference in the risk of high-grade thrombocytopenia between the carfilzomib and supportive therapy groups (*p* = 0.70), although smaller studies showed a higher incidence in the control group. Additionally, bortezomib was significantly more likely to cause high-grade thrombocytopenia compared to carfilzomib (*p* = 0.004), with grade 3 and 4 thrombocytopenia significantly more common in patients treated with bortezomib. The mechanisms of thrombocytopenia associated with bortezomib treatment were elucidated. Based on the studies in mice, the reduction in platelet count induced by bortezomib is not due to a cytotoxic effect on the bone marrow, as typical of most conventional chemotherapies, but rather because bortezomib reversibly impairs megakaryocyte function, which then manifests in platelet formation. In addition, patients with MM, who experience bortezomib-induced thrombocytopenia, should receive platelet transfusions rather than have treatment reduced or delayed due to thrombocytopenia.^26^ Comparison of different doses of carfilzomib showed no significant difference in the risk of high-grade thrombocytopenia between reduced and standard doses (*p* = 0.99), although there is a trend suggesting that higher doses may increase this risk.

Single-arm studies, or those comparing treatment with carfilzomib to other PIs, are more common because they are easier to conduct, often overshadowing RCTs comparing treatment with carfilzomib to the best available therapy alone. To address this, we used an alternative approach to analyze data from single-arm studies by contrasting them with scientific data from control groups in RCTs. However, due to potential differences in population selection criteria, careful interpretation is warranted, even if all included participants are MM patients.

Using this comparative approach, we found a 2.3-fold higher incidence of thrombocytopenia in patients treated with carfilzomib compared to patients receiving supportive therapy. Data from single-arm studies exhibit greater heterogeneity with wider prediction intervals, in contrast to more consistent incidence rates observed in RCTs. Moreover, the incidence of high-grade thrombocytopenia is significantly higher in patients treated with carfilzomib compared to patients receiving supportive therapy alone in both single-arm studies and RCTs. This emphasizes potential risks of thrombocytopenia associated with carfilzomib treatment.

## Comparison with other carfilzomib-induced AEs meta-analysis

Meta-analyses and systematic reviews have consistently identified hematological AEs, such as anemia, neutropenia, and thrombocytopenia, as common occurrences in MM patients undergoing carfilzomib treatment.^6–8^ Our meta-analysis examined the prevalence and severity of thrombocytopenia associated with carfilzomib treatment, emphasizing the importance of careful monitoring and management strategies during therapy. In contrast to our findings, Georgoulis et al. concluded in their overview of systemic reviews of the efficacy and safety of carfilzomib in the treatment of MM that the incidence of hematological AEs does not increase after the use of carfilzomib compared to other agents.^33^ Their review includes 14 published articles, including 9 systematic reviews and 5 meta-analyses. However, the publications often included data from the same studies (e.g., all publications included data from the ENDEAVOR study, 13 used data from the ASPIRE study, and 8 used data from the FOCUS study), which may lead to a multiplication of the outcomes and also cause a slight difference in the number of patients included in the meta-analyses.^33^ The majority of patients included were from ENDEAVOR and ASPIRE studies, where patients received median two cycles of MM treatment, which may result in lower thrombocytopenia incidence (Table S7 in Supplemental Material). In addition, the control group in Georgoulis et al. consisted of patients receiving supportive therapy only as well as patients receiving bortezomib treatment, in contrast to our meta-analysis, which had strict inclusion criteria to prevent duplication of data from the same participants. As a result, our study included a total of 9237 patients from 33 single-arm studies and 14 RCTs, which were divided into subgroups based on control groups, allowing a more comprehensive assessment of the AEs of carfilzomib. In particular, our analysis showed a significant difference in the risk of thrombocytopenia between carfilzomib and bortezomib treatments, highlighting the importance of considering concomitant drug therapy when evaluating the impact of carfilzomib on the occurrence of hematological AEs such as anemia, neutropenia, and thrombocytopenia.

Nevertheless, there is unanimous evidence from reviews and previous meta-analyses that treatment with carfilzomib increases the risk of cardiovascular adverse events (CVAEs), particularly the occurrence of all-grade hypertension and heart failure.^33–35^ In addition, carfilzomib has been associated with cardiotoxicity in two RCT-based meta-analyses.^34,35^ The first analysis included 24 clinical trials of carfilzomib use and collected information on 2594 patients. The estimated rate of CVAEs was 18.1% for all-grade and 8.2% for high-grade AEs. In addition, higher doses of carfilzomib were associated with a higher rate of AEs.^34^ A similar trend was observed in our RCTs based meta-analysis, in which the comparison of patients receiving standard and high doses of carfilzomib showed that higher doses of carfilzomib were more likely to cause thrombocytopenia. The second meta-analysis, with data from 4164 patients and 29 clinical trials, also confirmed that carfilzomib significantly increases the risk of cardiotoxicity. In addition, the ENDEAVOR study performed a direct comparison between carfilzomib and bortezomib treatment and found that carfilzomib was more likely to cause cardiotoxicity than bortezomib.^35^ However, our meta-analysis showed that, within the use of PIs, all-grade thrombocytopenia is more likely to be associated with carfilzomib treatment, while high-grade thrombocytopenia is usually a consequence of bortezomib treatment. These results are all based on data from RCTs, as most meta-analyses include RCTs and overlook the results of single-arm studies. In case of single-arm studies, the difference in the incidence of thrombocytopenia in patients treated with carfilzomib compared to the patients on supportive therapy could be a consequence of myelophthisis and bone marrow malfunction. Namely, patients who received more therapeutic regimens may also be more frequently treated with carfilzomib. Furthermore, the presence of cardiotoxicity, as a consequence of carfilzomib therapy, could be used as a “biomarker” for distinguishing between carfilzomib-induced thrombocytopenia versus the disease induced (e.g., myelopathies and stromal damage). However, the focus of our meta-analysis was on the occurrence of thrombocytopenia in RCTs presenting a direct comparison between patients on carfilzomib therapy and patients without this therapeutic drug. The data from multiple clinical trials suggest that clinicians should consider these safety limitations when treating MM patients with carfilzomib.

## Strengths and limitations

We consider that the dose of each therapeutic agent is an important factor in the occurrence of thrombocytopenia. With this in mind, we identify as the main strength of our meta-analysis the division of patients into subgroups based on the dose of carfilzomib received. In addition, appropriate control groups were established for individual analyses, based on either supportive therapy, treatment with bortezomib, or a lower carfilzomib dose. A predefined protocol registered in PROSPERO was used to screen clinical trials, so that the studies selected and the results obtained are considered transparent. The protocol involved using the Cochrane Risk of Bias Tool to assess the risk of bias for randomized control trials. However, this was not carried out because almost all of the RCTs obtained were either phase II or phase III clinical trials (carfilzomib registrational studies), and differences in the risk of bias within these studies were therefore not anticipated. A random effect model was used to evaluate the data obtained due to (1) population heterogeneity, namely the patient categories included, NDMM and RRMM, had different disease stages; (2) different supportive care approaches (drugs used and their dosage) in individual studies, potentially causing different adverse reactions that could be reported as additional AEs; and (3) different dosing of carfilzomib. Because the studies varied widely, we divided them into four groups according to the definition of the control group in RCTs and as single-arm studies. In each study, we examined the number of patients with thrombocytopenia (AE). Based on this approach, we selected keywords to search clinical trial databases and data registries. The retrieved literature was selected using the PRISMA approach, the results were ranked, and the selected studies were evaluated.

We are aware that the meta-analysis performed has its limitations in terms of the number of included studies, control groups and sample size. Namely, only 14 RCTs on carfilzomib treatment provided data on thrombocytopenia in MM patients, and some of these had no comparable control groups. For example, four RCTs had a control group of patients receiving supportive therapy only. This low number was expected given the severity of the condition, as it is unethical not to provide adequate treatment. In other three RCTs, the control group received reduced carfilzomib doses but had a smaller sample size (only 160 patients) than other carfilzomib study arms. Based on this, careful interpretation of the data is required as the number of participants may limit the power to assess clinically important outcomes.

## Conclusion

Carfilzomib is increasingly used in the treatment of patients with MM within many different therapeutic schemes. Several meta-analyses and systematic reviews have demonstrated the association between carfilzomib administration and cardiotoxicity, as well as hematological adverse effects. Herein, we specifically addressed the association between carfilzomib dosage and the occurrence of thrombocytopenia. Analysis of currently published data showed that treatment with carfilzomib likely increases the incidence of high-grade thrombocytopenia, which is important from the clinician’s treatment perspective. There is a clear correlation between the dosage concentration and AE occurrence. Surprisingly, when treatment with carfilzomib and bortezomib was compared, bortezomib was found to cause higher percentage of high-grade thrombocytopenia than carfilzomib. Nevertheless, with strict monitoring of patient clinical status and platelet count during PI treatment, we may prevent serious thrombocytopenia. For future reference, it will be of the great importance to define the level of platelet count that would serve as the predictive factor for the occurrence of severe thrombocytopenia.

## Supplemental Material

sj-docx-1-tah-10.1177_20406207241292517 – Supplemental material for A systematic review and meta-analysis of carfilzomib-associated thrombocytopenia as an adverse event in patients with multiple myeloma Supplemental material, sj-docx-1-tah-10.1177_20406207241292517 for A systematic review and meta-analysis of carfilzomib-associated thrombocytopenia as an adverse event in patients with multiple myeloma by Lara Smrdel, Igor Locatelli, Samo Zver and Martina Gobec in Therapeutic Advances in Hematology

## Declarations

**Ethics approval and consent to participate:** Not applicable.

**Consent for publication:** Not applicable.

**Availability of data and materials:** Data, data extraction templates, and analytic code are available on reasonable request.
