# Supplementary material for: A systematic review and meta-analysis of carfilzomib-associated thrombocytopenia as an adverse event in patients with multiple myeloma
Source: Ther Adv Hematol. 2024 Nov 13;15:20406207241292517. doi: 10.1177/20406207241292517 (PMC11558734; doi:10.1177/20406207241292517)
Supplement: sj-docx-1-tah-10.1177_20406207241292517 – Supplemental material for A systematic review and meta-analysis of carfilzomib-associated thrombocytopenia as an adverse event in patients with multiple myeloma [file sj-docx-1-tah-10.1177_20406207241292517.docx]

**​Supplemental Content**

**A systematic review and meta-analysis of carfilzomib-associated thrombocytopenia as an adverse event in patients with multiple myeloma**

Lara Smrdel^1^, Igor Locatelli^2^, Samo Zver^3^, Martina Gobec^1^*

^1^University of Ljubljana, Faculty of Pharmacy, Department of Clinical Biochemistry, Aškerčeva 7, SI-1000, Ljubljana, Slovenia

^2^University of Ljubljana, Faculty of Pharmacy, Department of Biopharmaceutics and Pharmacokinetics, Aškerčeva 7, SI-1000, Ljubljana, Slovenia

^3^University Medical Centre Ljubljana, Clinical Department of Haematology, Zaloška 7, SI-1000, Ljubljana, Slovenia

**Supplementary:**

- SC, Tables 1-7
- SC, Figures 1-6

**SC, Table S1.** Search strategy.

| **Database/ Register** | **Relational and logical operators** |
| --- | --- |
| PubMed | proteasome inhibitors [All Fields] AND (carfilzomib [All Fields] OR Kyprolis® [All Fields] OR PR-171 [All fields]) AND thrombocytopaenia [All Fields] AND multiple myeloma [All Fields] NOT review [Publication Type] |
| Web of science | ((((((TS=(carfilzomib)) OR ALL=(Kyprolis)) OR ALL=(PR-171)) AND ALL=(thrombocytopenia))) AND ALL=(multiple myeloma)) NOT DT=(Review) |
| Sci Finder | carfilzomib and thrombocytopenia |
| Cochrane Central Register of Controlled Trials | "proteasome inhibitor" in Title Abstract Keyword AND carfilzomib in Title Abstract Keyword OR PR-171 in Title Abstract Keyword AND "thrombocytopenia" in Title Abstract Keyword AND multiple myeloma in Title Abstract Keyword |
| ClinicalTrials.gov | carfilzomib OR PR-171 AND thrombocytopenia AND multiple myeloma |
| European Clinical Trials Register | carfilzomib AND multiple myeloma |

**SC, Table S2.** Characteristics of different studies included in meta-analysis. A. – single arm studies with results; B. – single arm studies with no results available.

| A. |  |  |  |  |  |  |
| --- | --- | --- | --- | --- | --- | --- |
| Author, Year of Publication | **Phase** | **Patients** | **Treatment Regimen** | **Carfilzomib Dosing [mg/m^2^]** | **Age [median]** | **NCT Number** |
| Badros 2013 | II | RRMM | Carf | 15 (days 1, 2, 8, 9, 15, 16) | 64 | NCT00721734 |
| Berdeja 2021 | I/II | RRMM | Pan, Carf | 20 (day 1) f/b 27 or 36 or 45 or 56 (days 8, 9, 15, 16) f/b 27 or 36 or 45 or 56 (days 1, 2, 8, 9, 15, 16) | 65.4 | NCT01496118 |
| Berenson 2014 | I/II | RRMM | Carf, IMiD | 20 (days 1, 2, 8, 9, 15, 16) f/b 27, 36 and 45 (days 1, 2, 8, 9, 15, 16) | 67 | NCT01365559 |
| Berenson 2016 | I/II | Progress. MM | Carf, Dex | 20 (day 1) f/b 70 (days 8, 15) | 67.5 | NCT01677858 |
| Bringhen 2014 | II | NDMM | Carf, Cyc, Dex | 20 (days 1, 2) f/b 36 (days 8, 9, 15, 16) f/b 36 (days 1, 2, 8, 9, 15, 16) | 71 | NCT01346787 |
| Bringhen 2017 | I/II | NDMM | Carf, Cyc, Dex | 20 (day 1) f/b 36 or 45 or 56 or 70 (days 8, 15) f/b 70 (days 1, 8, 15) | 72 | NCT01857115 |
| Camilleri 2021 | II | untreated MM | Carf, Cyc, Dex | 56 (days 1, 2, 8, 9, 15, 16) | 59 | NCT02315716 |
| Chari 2019 | Ib | RRMM | Dara, Carf, Dex | 20 (day 1) f/b 70 (days 8, 15) | 66 | NCT01998971 |
| Chari 2020 | Ib/II | RRMM | Ibr, Carf, Dex | 20 or 36 | 63.5 | NCT01962792 |
| Du 2021 | III | RRMM | Carf, Dex | 20 (days 1, 2) f/b 27 (days 8, 9, 15, 16) | 59.8 | NCT03029234 |
| Forsberg 2021 | II | NDMM | Carf, Len, Dex, Clar | 45 (days 1, 2, 8, 9, 15, 16) | 60 | NCT01559935 |
| Gasparetto 2022 | I/II | RRMM, NDMM | Seli, Dex, Btz, Carf, Clar | 56 or 70 (days 1, 8, 15) | NR | NCT02343042 |
| Gay 2021 | I/II | RRMM | Carf | 27 (days 1, 2, 15, 16) | 66 | NCT02056756 |
| Herndon 2013 | II | RRMM | Carf | 20 (days 1, 2, 8, 9, 15, 16) f/b 27 (days 1, 2, 8, 9, 15, 16) | 63 | - |
| Jakubowiak 2012 | Ib/II | NDMM | Carf, Len, Dex | MTD (days 1, 2, 8, 9, 15, 16) f/b MTD (days 1, 2, 15, 16) | 59 | NCT01029054 |
| Korde N 2015 | II | NDMM | Carf, Len, Dex | 20 (days 1, 2) f/b 36 (days 8, 9, 15, 16) f/b 36 (days 1, 2, 8, 9, 15, 16) | 60 | NCT01402284 |
| Lee 2021 | - | RRMM | Carf, Len, Dex | 20 (days 1, 2) f/b 27 (days 1, 2, 8, 9, 15, 16) f/b (days 1, 2, 15, 16) | 61 | - |
| Lendvai 2014 | II | RRMM | Carf | 56 (days 1, 2, 8, 9, 15, 16) | 63 | NCT01351623 |
| Moreau 2015 | I/II | NDMM | Carf, Mel, Pred | 20 (days 1, 2,8, 9, 22, 23, 29, 30) or 20 (days 1, 2) f/b 27 (days 8, 9, 22, 23, 29, 30) or 20 (days 1, 2) f/b 36 (days 8, 9, 22, 23, 29, 30) or 20 (days 1, 2) f/b 45 (days 8, 9, 22, 23, 29, 30) | 72 | NCT01279694 |
| Moreau 2021 | III | RRMM | Isa, Carf, Dex | 20 (days 1, 2) f/b 56 (days 8, 9, 15, 16) f/b 56 (days 1, 2, 8, 9, 15, 16) | 64 | NCT03275285 |
| NCT01057225 2017** | I/II | NDMM | Carf, Cyc, Thal, Dex | 20 (days 1, 2, 8, 9, 15, 16) f/b 27 (days 1, 2, 8, 9, 15, 16) or 36 (days 1, 2, 8, 9, 15, 16) | 62.5 | NCT01057225 |
| NCT00884312 2017** | II | MM | Carf | 11 - 27 (days 1, 2, 8, 9, 15, 16) and 36 - 56 (days 1, 2, 8, 9, 15, 16) | 63 | NCT00884312 |
| NCT01775553 2016** | II | RRMM | Carf | 20 (days 1,2) or 56 (days 1,2) f/b 56 (day 8) | 62.9 | NCT01775553 |
| NCT01842308 2020** | I/II | pl. cell lymph. | Carf, Mel | 56 (days 1, 2, 5, 6) | 60 | NCT01842308 |
| Quach 2021 | III | RRMM | Carf, Dex | 20 (days 1, 2) f/b 56 (days 8, 9, 15, 16) f/b 56 (days 1, 2, 8, 9, 15, 16) | 64.3 | NCT03158688 |
| Schroeder 2019 | I/II | RRMM | Carf, PLD, Dex | 56 (days 1, 2, 8, 9, 15, 16) f/b 56 (days 1, 8, 15, 22) | 65 | NCT01246063 |
| Shah 2015 | I/II | RRMM | Carf, Pom, Dex | 20/27 (days 1, 2, 8, 9, 15, 16) | 64 | NCT01464034 |
| Shinsuke 2019 | I/II | RRMM | Carf | 15/20 (days 1, 2, 8, 9, 15, 16) or 20 (days 1,2) f/b 27 (days 8, 9, 15, 16) | NR | - |
| Tremblay 2021 | II | RRMM | Seli, Dex | NR | 63.9 | NCT02336815 |
| Uysal 2018 | - | RRMM | Carf, Dex | 20 (days 1, 2, 8, 9, 15, 16) f/b 27 (days 1, 2, 8, 9, 15, 16) | 62 | - |
| Venner 2021 | II | RMM | Carf, Cyc, Dex | 20 (day 1) f/b 70 (days 1, 8, 15) | 66 | NCT02597062 |
| B. |  |  |  |  |  |  |
| Author, Year of Publication | **Phase** | **Patients** | **Treatment Regimen** | **Carfilzomib Dosing [mg/m^2^]** | **Age [median]** | **NCT Number** |
| Bertamini 2022 | II | NDMM | Carf, Cyc, Dex, Len | 36 (days 1, 2, 8, 9, 15, 16) | 57 | NCT02203643 |
| Costa 2018 | I/IIa | RRMM | Carf, Mel | MTD (days 2, 3) | 58 | NCT01690143 |
| Leleu 2019 | III | RRMM | Carf, Dex, Len | Once weekly group 30 mins 🡪 20 (day 1) f/b 56 (days 8, 15) vs. twice-weekly group 10 mins 🡪 20 (days 1, 2) f/b 27 (days 8, 9, 15, 16) | NR | NCT03859427 |
| Leypoldt 2022 | II |  | Isa, Carf, Len, Dex | 20 (days 1, 2) f/b 36 (days 8, 9, 15, 16) f/b (days 1, 2, 8, 9, 15, 16) | 58 | NCT03104842 |

* List of abbreviations: RRMM – relapse/refractory multiple myeloma, NDMM – newly diagnosed multiple myeloma, NR – no results, Carf – carfilzomib, Dex – dexamethasone, Btz – bortezomib, Len – lenalidomide, Mel - melphalan, Pred - prednisone, Cyclo – cyclophosphamide, MTD – maximum tolerated dose (exact dosing regimen not reported).

** Data not published yet; available on ClinicalTrials.gov.

**SC, Table S3.** Incidence of thrombocytopenia in single-arm studies.

| Author, Year of Publication | Phase | Total N | Carfilzomib Treatment | | |
| --- | --- | --- | --- | --- | --- |
|  |  |  | **all grade** | **high grade** | **N _scr._** |
| Badros 2013 | II | 50 | 25 | 10 | 50 |
| Berdeja 2021 | I/II | 80 | 44 | 0 | 61 |
| Berenson 2014 | I/II | 39 | 30 | 9 | 39 |
| Berenson 2016 | I/II | 116 | 31 | 2 | 89 |
| Bertamini 2022 | II | 477 | NR | NR | - |
| Bringhen 2014 | II | 58 | 21 | 2 | 56 |
| Bringhen 2017 | I/II | 54 | 18 | 4 | 54 |
| Camilleri 2021 | II | 281 | 7 | NR | 8 |
| Chari 2020 | Ib/II | 84 | 45 | 1 | 84 |
| Costa 2018 | I/IIa | 45 | NR | NR | - |
| Du 2021 | III | 126 | 72 | 5 | 123 |
| Forsberg 2021 | II | 74 | 65 | 9 | 72 |
| Gasparetto 2022 | I/II | 518 | 72% | 47% | - |
| Gay 2021 | I/II | 63 | 23 | 14 | 63 |
| Herndon 2013 | II | 526 | 314 | 123 | 526 |
| Jakubowiak 2012 | Ib/II | 53 | 36 | NR | 53 |
| Korde 2015 | II | 45 | 44 | 2 | 45 |
| Lee 2021 | - | 55 | 26 | 16 | 55 |
| Leleu 2019 | III | 454 | NR | NR | - |
| Chari 2019 | Ib | 242 | 57 | 26 | 85 |
| Lendvai 2014 | II | 44 | 17 | 5 | 44 |
| Leypoldt 2022 | II | 246 | > 10% | > 10% | - |
| Moreau 2015 | I/II | 68 | 58 | 19 | 68 |
| Moreau 2021 | III | 302 | 107 | 29 | 122 |
| NCT01057225 2017* | I/II | 64 | 30 | NR | 64 |
| NCT00884312 2017* | II | 101 | 16 | NR | 91 |
| NCT01775553 2016* | II | 13 | 7 | NR | 13 |
| NCT01842308 2020* | I/II | 50 | 50 | NR | 50 |
| Quach 2021 | III | 466 | 46 | 1 | 153 |
| Schroeder 2019 | I/II | 40 | 35 | 40% | 40 |
| Shah 2015 | I/II | 136 | 10 | 7 | 32 |
| Shinsuke 2019 | I/II | 50 | 34 | 13 | 46 |
| Tremblay 2021 | II | 202 | 149 | 8 | 202 |
| Uysal 2018 | - | 21 | 8 | 3 | 21 |
| Venner 2021 | II | 76 | 34 | 15 | 75 |

* Data not published yet; available on ClinicalTrials.gov.

*N scr. = number of patients screened for thrombocytopenia occurrence*

**SC, Table S4.** Supportive therapy dosage data.

| Authors, Study Name, Phase | Treatment Regimen | Dexamethasone Dosing [mg] | Prednisone Dosing [mg/m^2^] | Lenalidomide Dosing [mg] | Pomalidomide Dosing [mg] | Cyclophosphamide Dosing [mg/m^2^] | Melphalan Dosing [mg/m^2^] | NCT number |
| --- | --- | --- | --- | --- | --- | --- | --- | --- |
| Ailawadhi 2020  S1304, II | Carf, Dex | 20 mg (days 1, 2, 8, 9, 15, 16) | - | - | - | - | - | NCT01903811 |
| Brown 2016  MUK five, II | Carf, Btz, Dex, Cyc | 40 mg weekly | - | - | - | 500 mg PO (days 1, 8, 15) | - | ISRCTN17354232 |
| Dimopoulos 2017  ASPIRE, III | Carf, Len, Dex | 40 mg (days 1, 8, 15, 22) | - | 25 mg (days 1– 21) | - | - | - | NCT01080391 |
| Facon 2019  CLARION, III | Carf, Btz, Mel, Pred | - | 60 mg/m² (days 1–4) | - | - | - | 9 mg/m² (days 1–4) | NCT01818752 |
| Gregersen 2021  CARFI, II | Carf, Cyc, Dex | 20 mg (days 1, 2, 8, 9, 15, 16) | - | - | - | 300 mg/m^2^ (days 1, 8, 15) | - | NCT02572492 |
| Hajek 2017  FOCUS, III | Carf | - | - | - | - | - | - | NCT01302392 |
| Jackson 2021  Myeloma XI+, III | Carf, Len, Dex, Cyc | 40 mg (days 1–4, 12–15) or 20 mg (days 1, 2, 4, 5, 8, 9, 11, 12) | - | 25 mg (days 1–21) | - | 500 mg (days 1, 8) | - | ISRCTN49407852 |
| Jagannath 2012 & Siegel 2012  PX-171-003-A1, II | Carf | - | - | - | - | - | - | NCT00511238 |
| Kumar 2020  EDURANCE, III | Carf, Btz, Dex, Len | 20 mg (days 1, 2, 4, 5, 8, 9, 11, 12) | - | 25 mg (days 1–14) | - | - | - | NCT01863550 |
| Ludwig 2019  ENDEAVOR, III | Carf, Btz, Dex | 20 mg (days 1, 2, 8, 9, 15, 16, 22, 23) | - | - | - | - | - | NCT01568866 |
| Mehta 2021  - | Carf, Pom, Dex | 20 mg weekly | - | - | 4mg (days 1–21) | - | - | - |
| Moreau 2019  ARROW, III | Carf, Dex | 40 mg (days 1, 8, 15, 22) f/b (days 1, 8, 15) | - | - | - | - | - | NCT02412878 |
| Vij 2012  PX-171-004, II | Carf | - | - | - | - | - | - | NCT00530816 |
| Wang 2013  PX-171-006, II | Carf, Len, Dex | 40 mg (days 1, 8, 15, 22) | - | 10, 15, 20, or 25 mg (days 1-21) | - | - | - | NCT00603447 |

* List of abbreviations: Carf – carfilzomib, Dex – dexamethasone, Btz – bortezomib, Len – lenalidomide, Mel - melphalan, Pred - prednisone, Cyclo – cyclophosphamide.

**SC, Table S5.** Incidence of neutropenia.

| Authors, Study Name, Phase | Treatment Regimen | Study-arm  Neutropenia | | N _scr._ | Control-arm  Neutropenia | | N _scr._ | NCT number |
| --- | --- | --- | --- | --- | --- | --- | --- | --- |
|  |  | **All-grade** | **High-grade** |  | **All-grade** | **High-grade** |  |  |
| Ailawadhi 2020  S1304, II | Carf, Dex | - | - | 66 | - | - | 57 | NCT01903811 |
| Brown 2016  MUK five, II | Carf, Btz, Dex, Cyc | 76 | 22 | 196 | 44 | 21 | 96 | ISRCTN17354232 |
| Dimopoulos 2017  ASPIRE, III | Carf, Len, Dex | 157 | 4 | 392 | 133 | 5 | 389 | NCT01080391 |
| Facon 2019  CLARION, III | Carf, Btz, Mel, Pred | 121 | 4 | 474 | 121 | 4 | 470 | NCT01818752 |
| Gregersen 2021  CARFI, II | Carf, Cyc, Dex | 24 | 3 | 82 | 23 | 4 | 86 | NCT02572492 |
| Hajek 2017  FOCUS, III | Carf | 22 | 1 | 157 | 23 | 3 | 153 | NCT01302392 |
| Jackson 2021  Myeloma XI+, III | Carf, Len, Dex, Cyc | 172 | 84 | 511 | 148 | 91 | 518 | ISRCTN49407852 |
| Jagannath 2012 & Siegel 2012  PX-171-003-A1, II | Carf | 10 | - | 46 | 48 | - | 266 | NCT00511238 |
| Kumar 2020  EDURANCE, III | Carf, Btz, Dex, Len | - | - | 526 | - | - | 527 | NCT01863550 |
| Ludwig 2019  ENDEAVOR, III | Carf, Btz, Dex | 27 | 1 | 463 | 26 | 0 | 456 | NCT01568866 |
| Mehta 2021  - | Carf, Pom, Dex | 11 | - | 30 | 5 | - | 39 | - |
| Moreau 2019  ARROW, III | Carf, Dex | 20 | - | 238 | 23 | - | 235 | NCT02412878 |
| Vij 2012  PX-171-004, II | Carf | 27 | 0 | 94 | 20 | 2 | 70 | NCT00530816 |
| Wang 2013  PX-171-006, II | Carf, Len, Dex | 12 | 0 | 20 | 26 | 1 | 64 | NCT00603447 |

N scr. = number of patients screened for neutropenia occurrence

**SC, Table S6.** Incidence of thrombocytopenia.

| Authors, Study Name, Phase | Treatment Regimen | Study-arm  Thrombocytopenia | | | N _scr._ | Control-arm  Thrombocytopenia | | N _scr._ | NCT number |
| --- | --- | --- | --- | --- | --- | --- | --- | --- | --- |
|  |  | **All-grade** | **High-grade** | |  | **All-grade** | **High-grade** |  |  |
| Ailawadhi 2020  S1304, II | Carf, Dex | 32 | 4 | 66 | | 36 | 7 | 57 | NCT01903811 |
| Brown 2016  MUK five, II | Carf, Btz, Dex, Cyc | 167 | 22 | 196 | | 65 | 21 | 96 | ISRCTN17354232 |
| Dimopoulos 2017  ASPIRE, III | Carf, Len, Dex | 115 | 5 | 392 | | 94 | 4 | 389 | NCT01080391 |
| Facon 2019  CLARION, III | Carf, Btz, Mel, Pred | 139 | 7 | 474 | | 166 | 14 | 470 | NCT01818752 |
| Gregersen 2021  CARFI, II | Carf, Cyc, Dex | 24 | 0 | 82 | | 18 | 2 | 86 | NCT02572492 |
| Hajek 2017  FOCUS, III | Carf | 70 | 3 | 157 | | 54 | 5 | 153 | NCT01302392 |
| Jackson 2021  Myeloma XI+, III | Carf, Len, Dex, Cyc | 256 | 43 | 511 | | 124 | 9 | 518 | ISRCTN49407852 |
| Jagannath 2012 & Siegel 2012  PX-171-003-A1, II | Carf | 23 | 0 | 46 | | 103 | 5 | 266 | NCT00511238 |
| Kumar 2020  EDURANCE, III | Carf, Btz, Dex, Len | 26 | 3 | 526 | | 11 | 4 | 527 | NCT01863550 |
| Ludwig 2019  ENDEAVOR, III | Carf, Btz, Dex | 163 | 6 | 463 | | 133 | 9 | 456 | NCT01568866 |
| Mehta 2021  - | Carf, Pom, Dex | 9 | 4 | 30 | | 6 | 1 | 39 | - |
| Moreau 2019  ARROW, III | Carf, Dex | 62 | 5 | 238 | | 45 | 5 | 235 | NCT02412878 |
| Vij 2012  PX-171-004, II | Carf | 31 | 1 | 94 | | 19 | 0 | 70 | NCT00530816 |
| Wang 2013  PX-171-006, II | Carf, Len, Dex | 8 | 0 | 20 | | 22 | 1 | 64 | NCT00603447 |

*N scr. = number of patients screened for thrombocytopenia occurrence*

***SC, Table S7.***  *Characteristics of patients from included RCTs, like treatment regimens, median age, line of treatment, ISS disease stage, ECOG/WHO performance status and FISH analysis.*

| Authors | Patients | Treatment Regimen | Median Age | Line of Treatment | Total N | ISS Disease Stage | | | | ECOG Performance Status | | | | Cytogenetic/FISH Analysis [patients] |
| --- | --- | --- | --- | --- | --- | --- | --- | --- | --- | --- | --- | --- | --- | --- |
|  |  |  |  |  |  | **I** | **II** | **III** | **NR** | **0** | **1** | **>2** | **NR** |  |
| Ailawadhi 2020 | RRMM | Carf, Dex | ≥ 65 | 1 – 6 | 143 | 42 | 49 | 30 | NR | - | | | | NR |
| Brown 2016 | RRMM | Carf, Btz, Dex, Cyc | 67 | 0 | 292 | 154 | 101 | 44 | 1 | 168 | 113 | 15 | 4 | 102 with HR |
| Dimopoulos 2017 | RMM | Carf, Len, Dex | 63,9 | 1 – 3 | 792 | - | | | | 717 | | 75 | - | 100 with HR |
| Facon 2019 | NDMM | Carf, Btz, Mel, Pred | 71,7 | 0 | 955 | 178 | 414 | 362 | 1 | - | | | | 121 with HR |
| Gregersen 2021 | RMM | Carf, Cyc, Dex | 62 | ≥ 1 | 200 | 106 | 65 | 18 | 11 | 120 | 63 | 8 | 9 | 39 with HR |
| Hajek 2017 | RRMM | Carf | 64,4 | ≥ 3 | 315 | 46 | 73 | 122 | 74 | 80 | 171 | 64 | - | 51 with HR |
| Jackson 2021 | NDMM | Carf, Len, Dex, Cyc | 62 | 0 | 1029 | 331 | 392 | 239 | 94 | - | | | | 179 with HR |
| Jagannath 2012 & Siegel 2012 | RRMM | Carf | 60-65 | ≥ 2 | 312 | 76 | 102 | 81 | NR | 69 | 162 | 35 | NR | 75 with genetic predisposition |
| Kumar 2020 | NDMM | Carf, Btz, Dex, Len | 64,5 | 0 | 1087 | 397 | 387 | 297 | 3 | 453 | 519 | 115 | - | 255 with abnormal cytogenetics |
| Ludwig 2019 | RMM | Carf, Btz, Dex | 65 | 1 – 3 | 929 | 409 | 520 | | NR | 453 | 414 | 62 | - | NR |
| Mehta 2021 | RRMM | Carf, Pom, Dex | 57 | ≥ 1 | 69 | 13 | 17 | 28 | 11 | - | | | | 25 FISH positive |
| Moreau 2019 | RRMM | Carf, Dex | 66 | 2 – 3 | 478 | 191 | 156 | 115 | 7 | 232 | 236 | 1 | - | 80 with HR |
| Vij 2012 | RMM | Carf | 65 | 1 – 3 | 164 | 94 | | 22 | 13 | 52 | 77 | | - | 19 with poor cytogenetic markers |
| Wang 2013 | RMM | Carf, Len, Dex | 61,9 | 1 – 3 | 84 | - | | | | 33 | 46 | 5 | - | 22 with HR |

** List of abbreviations: ISS – international staging system, ECOG – Eastern cooperative oncology group, RRMM – relapse/refractory multiple myeloma, NDMM – newly diagnosed multiple myeloma, NR – no results, Carf – carfilzomib, Dex – dexamethasone, Btz – bortezomib, Len – lenalidomide, Mel - melphalan, Pred - prednisone, Cyclo – cyclophosphamide, HR – high risk.*

**SC, Supplementary Figures 1–6**


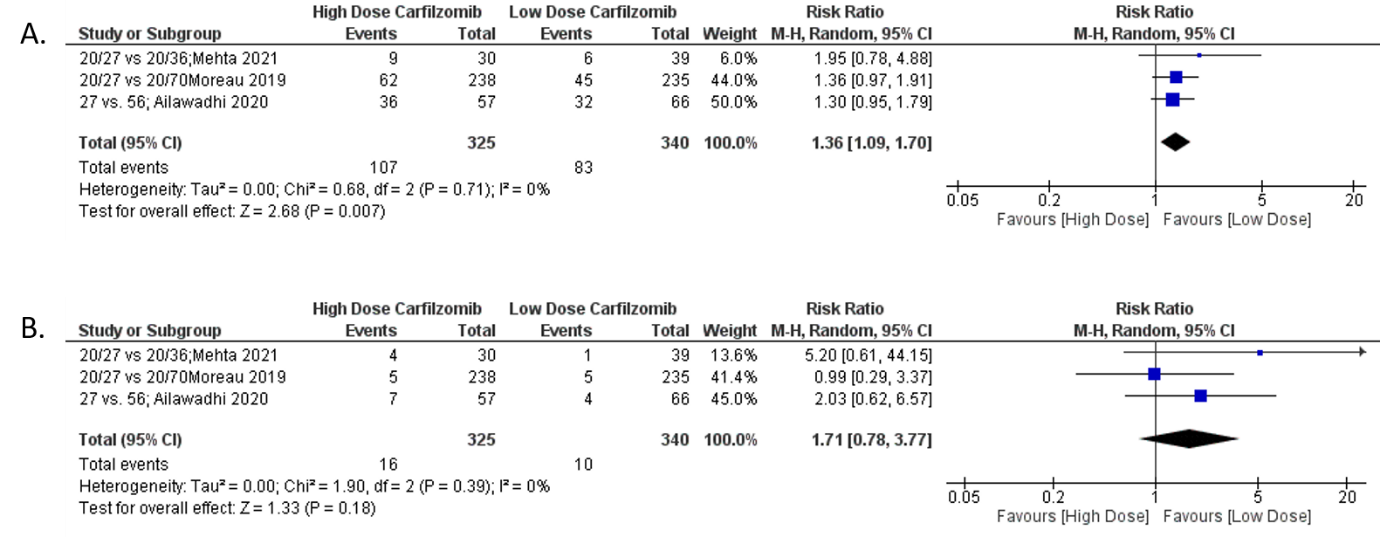


**SC, Figure S1.** Forest plots of all-grade and high-grade thrombocytopenia (high carfilzomib doses vs. standard carfilzomib doses); (A) all-grade thrombocytopenia; (B), high-grade thrombocytopenia.


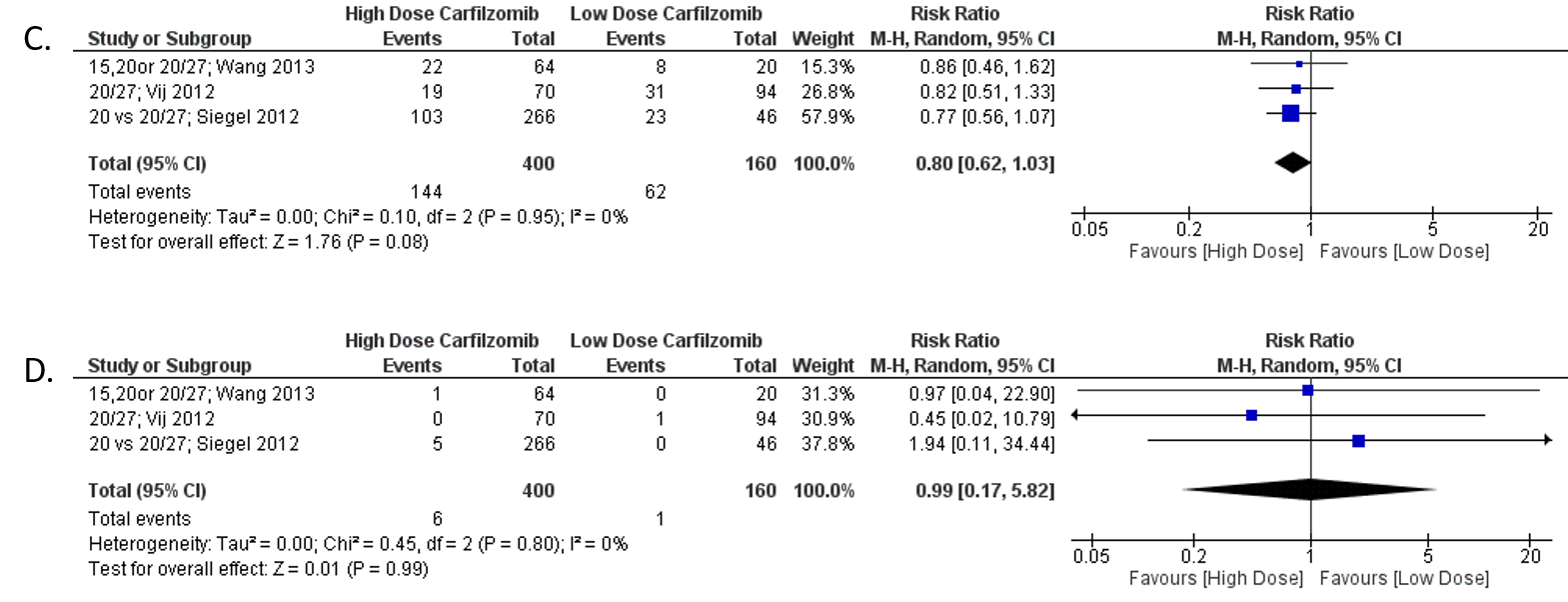


**SC, Figure S2.** Forest plots of all-grade and high-grade thrombocytopenia (standard carfilzomib doses (defined as high) vs. reduced carfilzomib doses (defined as low)); (C) all-grade thrombocytopenia; (D), high-grade thrombocytopenia.


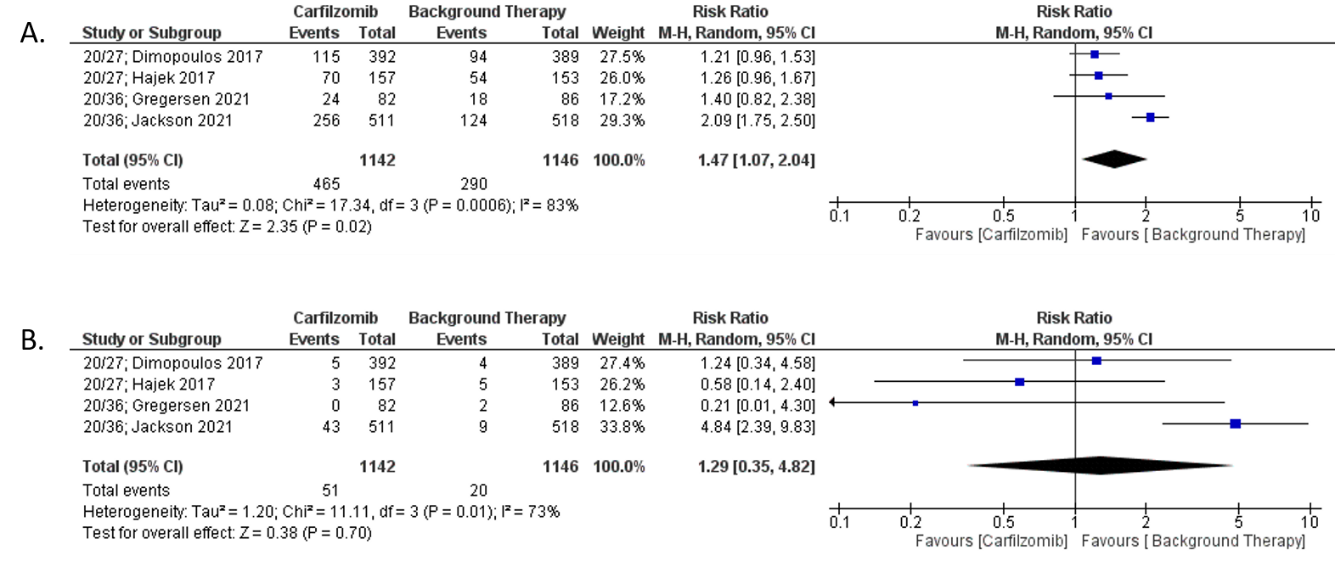


**SC, Figure S3.** Forest plots of all-grade and high-grade thrombocytopenia (standard carfilzomib doses vs. supportive therapy only); (A) all-grade thrombocytopenia; (B), high-grade thrombocytopenia.


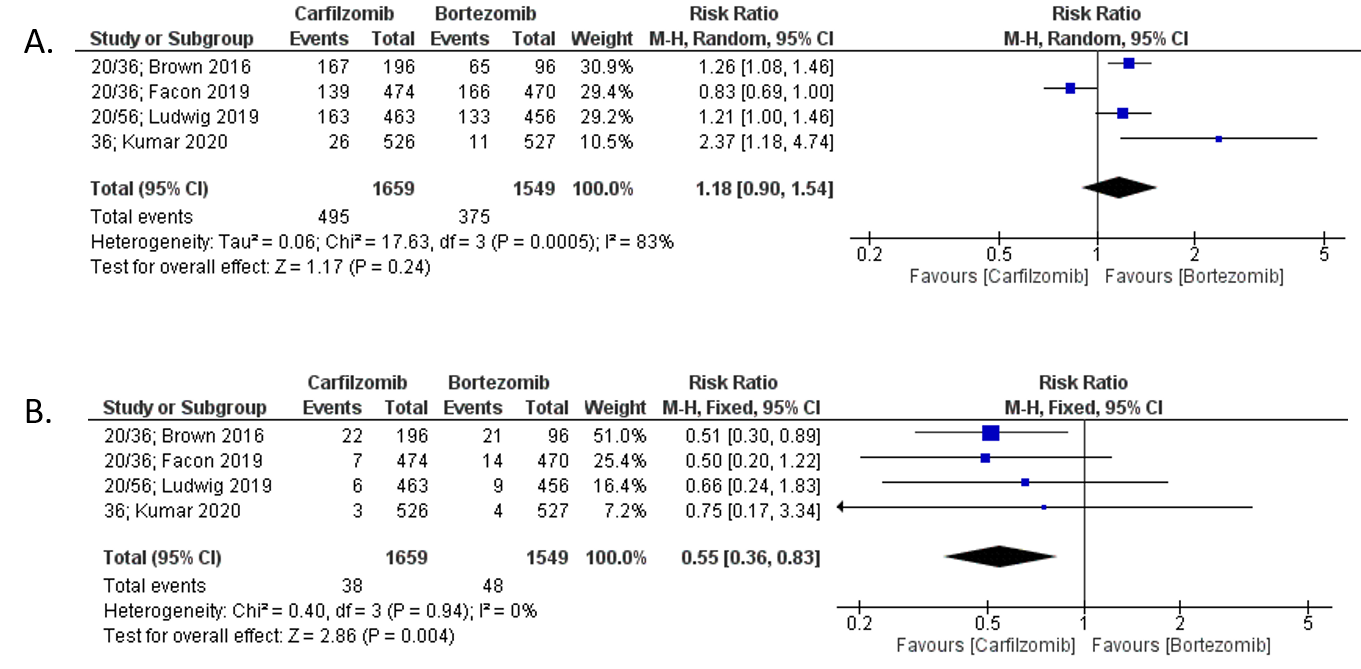


**SC, Figure S4.** Forest plots of all-grade and high-grade thrombocytopenia (standard carfilzomib doses vs. standard bortezomib doses); (A) all-grade thrombocytopenia; (B), high-grade thrombocytopenia.


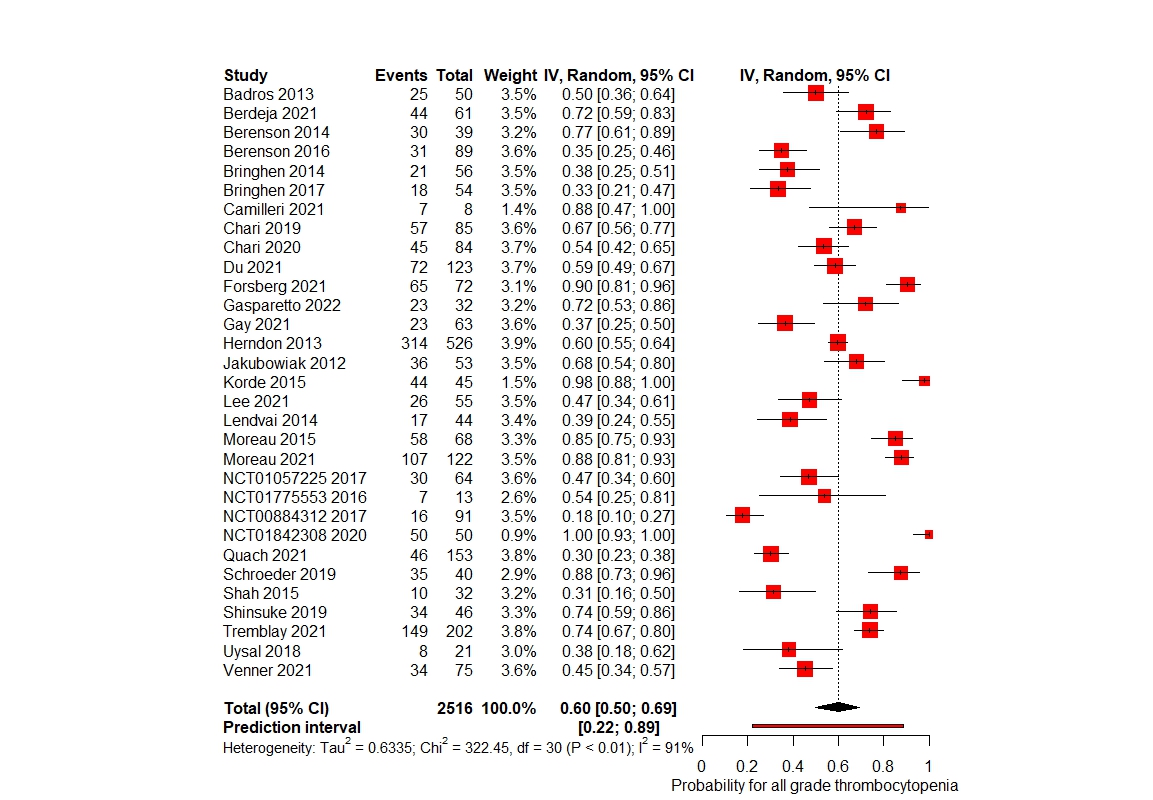


**SC, Figure S5.** Forest plots of all-grade thrombocytopenia in single-arm studies.


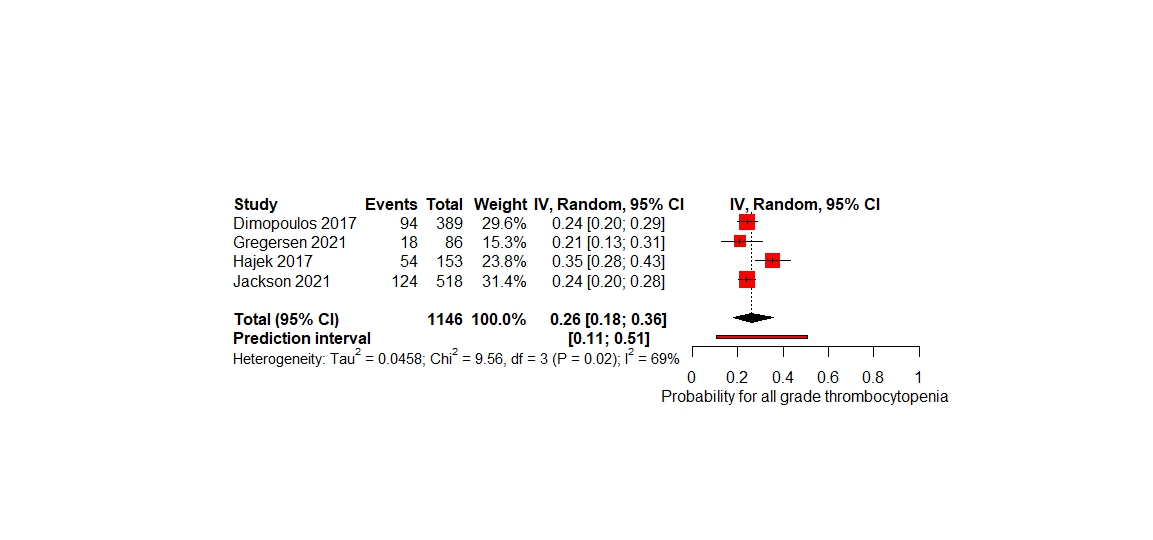


**SC, Figure S6.** Forest plots of all-grade thrombocytopenia of supportive therapy treatment in 4 RCTs.
